# Epicardial adipose tissue in cardiovascular diseases: a potential therapeutic target

**DOI:** 10.3389/fcvm.2026.1817940

**Published:** 2026-06-18

**Authors:** Zhaoting Gong, Yuyan Xiong

**Affiliations:** 1 Center for Coronary Artery Disease, Division of Cardiology, Beijing Anzhen Hospital, Capital Medical University, Beijing, China; 2 Department of Cardiology, Union Hospital, Tongji Medical College, Huazhong University of Science and Technology, Wuhan, China; 3 Hubei Key Laboratory of Biological Targeted Therapy, Union Hospital, Tongji Medical College, Huazhong University of Science and Technology, Wuhan, China; 4 Hubei Provincial Engineering Research Center of Immunological Diagnosis and Therapy for Cardiovascular Diseases, Union Hospital, Tongji Medical College, Huazhong University of Science and Technology, Wuhan, China

**Keywords:** biomarker, cardiovascular disease, epicardial adipose tissue, extracellular vesicle, therapeutic target

## Abstract

Epicardial adipose tissue (EAT) is a distinct adipose depot located between the myocardium and the visceral pericardium. It is distinct from other visceral adipose tissue due to its anatomical position, vicinity to the heart, and its unique secretomic profile. EAT is increasingly recognized for its association with cardiovascular events and is being investigated as a therapeutic target for cardiovascular diseases (CVDs). This review provides an overview of the anatomy and physiology of EAT, discusses different imaging techniques for EAT assessment, explores the association between EAT and CVDs, and also describes its clinical application as a biomarker and potential therapeutic target.

## 1 Introduction

Despite significant progress in risk prevention, treatment, and control, cardiovascular diseases (CVDs) remain the leading cause of mortality and disability worldwide (1). Smoking, obesity, hypertension, diabetes mellitus, and dyslipidemia are widely recognized risk factors for CVDs (2), with obesity emerging as a global healthcare challenge due to its contribution to CVD-related mortality (3). Increasing interest in the relationship between epicardial adipose tissue (EAT) and CVDs has shifted cardiometabolic research from a broad focus on general obesity toward a more detailed investigation of organ-specific adiposity (4). EAT, a distinct and multifaceted adipose depot, lies between the myocardium and the visceral pericardium, and exhibits unique properties. Its anatomical position, proximity to the heart, and its unique profile distinguish it from other visceral adipose tissue (5). EAT plays a dual role: its metabolic, thermogenic, and mechanical properties provide protection to the adjacent myocardium, whereas its paracrine secretion of proinflammatory and profibrotic cytokines can be detrimental under pathological conditions (6). Given its contiguity to the heart, EAT might crosstalk with the myocardium in a paracrine or vasocrine manner. Given its unique structural and functional features, EAT is considered to be associated with the progression and development of CVDs, including coronary artery disease (CAD), atrial fibrillation (AF), and heart failure (HF) (7, 8), although the precise mechanisms remain an area of active investigation. In recent studies, various non-invasive imaging modalities, such as echocardiography, computed tomography (CT), and magnetic resonance imaging (MRI), were employed to assess EAT and evaluate CVD risk. Nevertheless, the specific molecular pathways connecting EAT to CVDs have not yet been fully elucidated. Recently, the role of extracellular vesicles (EVs) in physiological and pathological conditions has attracted attention (9). As carriers of proteins, lipids, and nucleic acids, EVs play a vital role in intercellular communication, which may contribute to the progression of CVDs, including atherosclerosis (10), AF (11), and HF (12). While the paracrine role of EAT in CVDs is challenging to assess directly, the unique signature of EAT-derived EVs sheds light on how this tissue may influence the development and progression of CVDs. With growing understanding of EAT biology, EAT is increasingly regarded not only as a possible indicator of CVDs but also as a promising therapeutic target.

**Figure 1:**
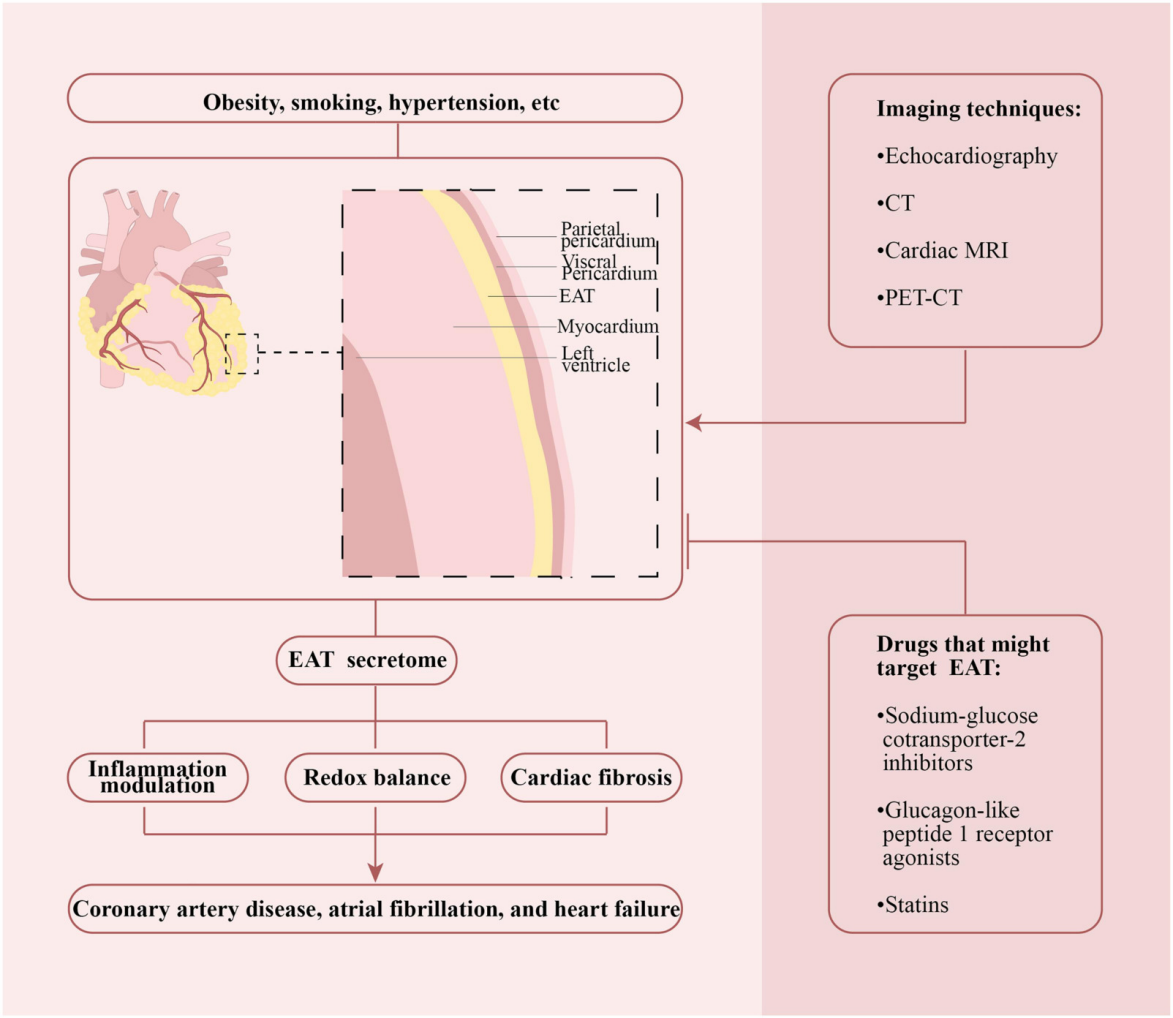
Potential role of epicardial adipose tissue in cardiovascular diseases. EAT is a unique fat depot located between the myocardium and the visceral pericardium. The EAT secretome may contribute to the pathogenesis of CVDs, such as CAD, AF, and HF, via paracrine mechanisms. Various imaging techniques are employed to evaluate EAT thickness or volume. Pharmacological agents such as sodium-glucose cotransporter 2 (SGLT2) inhibitors, glucagon-like peptide 1 receptor (GLP1R) agonists, and statins may target EAT, leading to cardioprotective effects.

This review provides an overview of the anatomy, physiology, and assessment of EAT, with particular emphasis on the emerging role of EAT-derived EVs, with insights into the role EAT in CVDs. We further discuss the clinical applications of EAT as a biomarker and a potential therapeutic target (Figure 1).

## 2 EAT overview

### 2.1 Anatomy and physiology of EAT

Cardiac adipose tissue can be classified into two distinct compartments based on their anatomical locations: EAT and pericardial adipose tissue (PAT). EAT, which originates from the splanchnopleuric mesoderm and is supplied by coronary artery branches, is located between the myocardium and the pericardial visceral layer. PAT is a layer of adipose tissue positioned between the visceral pericardium and the parietal pericardium. In addition, EAT accounts for approximately 80% of the heart's surface area and 20% of its total weight (13).

EAT is a specialized adipose depot that exhibits features of white adipose tissue while also possessing characteristics of brown and beige fat (14). Notably, EAT is directly contiguous with the myocardium, lacking any intervening muscle fascia. This anatomic proximity, combined with the absence of a fascial barrier and the presence of a shared microcirculation, allows for potential crosstalk with the myocardium through paracrine mechanisms.

Under physiological conditions, EAT exerts cardioprotective effects. It has a high capacity for the release and uptake of free fatty acids (FFAs), thereby supplying FFAs to the contiguous myocardium to provide energy for the myocardium and acting as a buffer to shield the heart from excessive FFA exposure (15). Epicardial fat also displays properties similar to brown adipose tissue (BAT), providing thermal support to the myocardium (16). Furthermore, epicardial fat provides a protective role for the heart and coronary arteries by mitigating mechanical deformation caused by arterial cardiac contractions and pulse waves (17). EAT also serves as a source of anti-inflammatory and anti-atherogenic molecules, such as adiponectin and adrenomedullin (18, 19), which exhibit cardioprotective effects.

Under pathological conditions, however, the function of EAT is altered. For instance, the expression levels of adiponectin in EAT are markedly lower in patients with CAD or HF than in healthy individuals (18, 20). In addition, dysfunctional atrium-infiltrating EAT is enriched in genes encoding arrhythmogenic proteins associated with oxidative phosphorylation, muscular contraction, and calcium signaling, rendering EAT an independent risk factor for the development and recurrence of AF following catheter ablation (21).

### 2.2 Assessment of EAT

**Figure 2:**
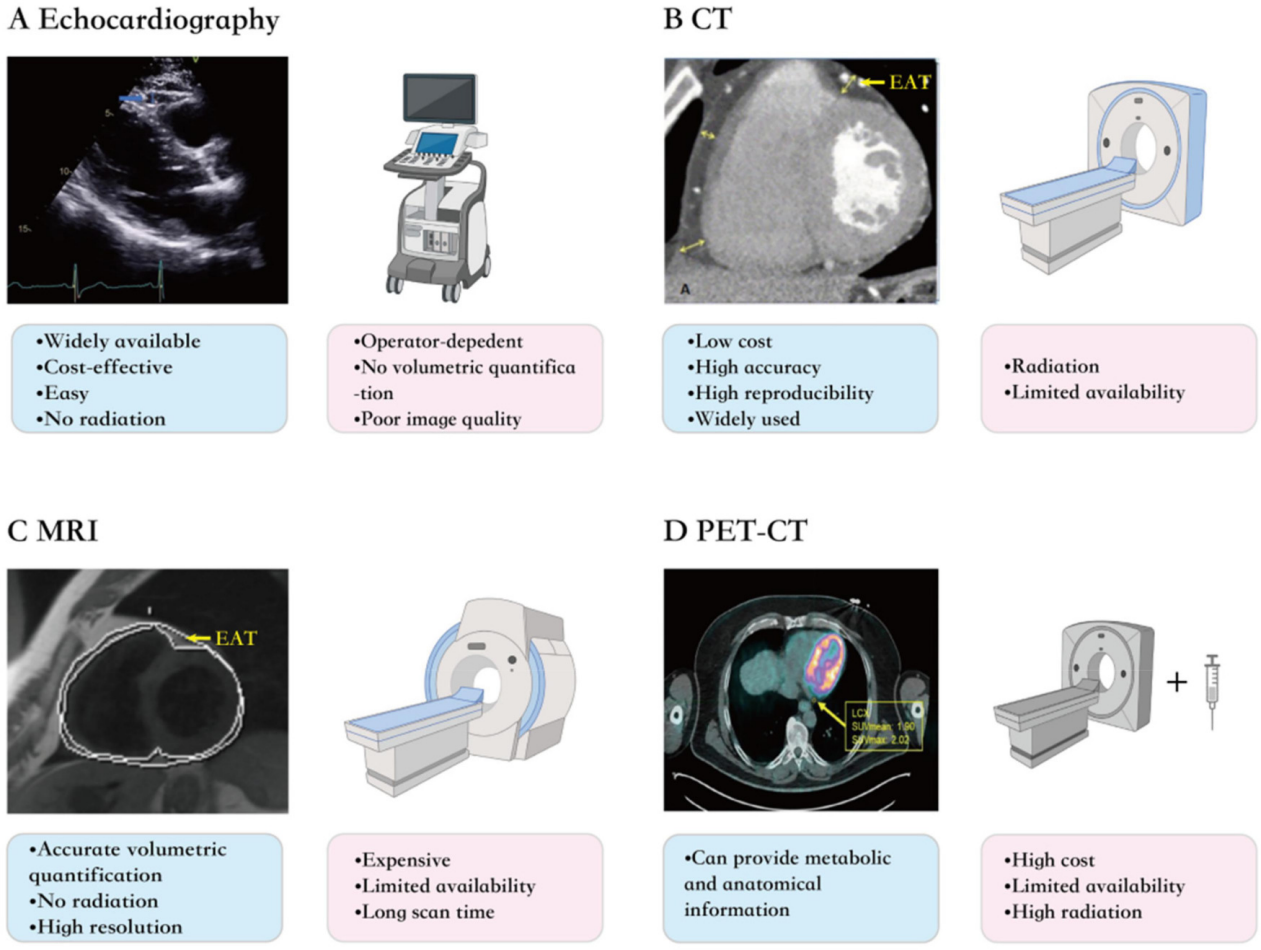
Imaging techniques for the evaluation of EAT. The relative advantages (blue boxes) and limitations (red boxes) of several commonly used clinical imaging techniques are highlighted. (A) Epicardial fat is characterized as the echo-free space located between the myocardium and the visceral pericardium. Echocardiography image reproduced from “EAT in parasternal long axis view” by Fabián Islas, Eva Gutiérrez, Victoria Cachofeiro, Ernesto Martínez-Martínez, Gema Marín, Carmen Olmos, Irene Carrión, Sandra Gil, Patricia Mahía, Miguel Ángel Cobos, Alberto de Agustín and María Luaces 1, licensed under CC BY. Echocardiography is a commonly accessible and safe technique, though it is operator-dependent. (B) CT imaging has high accuracy and reproducibility, but it involves radiation exposure. Adapted from “A) Epicardial fat thickness (yellow arrows)” by Muhammed Bora Demircelik, Omer Caglar Yilmaz, Ozgul Malcok Gurel, Yusuf Selcoki, Inci Asli Atar, Alper Bozkurt, Kayihan Akin and Beyhan Eryonucu, licensed under CC BY-NC. (C) MRI can provide accurate volumetric quantification and avoid radiation exposure, but requires longer acquisition times and higher costs. Adapted from “Difference in EAT mass in healthy controls and patients with CHF. Volumetric measurement of EAT outlining the contours of EAT in end-diastolic images of short axis covering the left and right ventricle in a healthy control with normal EAT mass (Panel A)” by Christina Doesch, Dariusch Haghi, Stephan Flüchter, Tim Suselbeck, Stefan O Schoenberg, Henrik Michaely, Martin Borggrefe and Theano Papavassiliu, licensed under CC BY. (D) PET-CT can provide both anatomical and metabolic information (such as inflammatory activity). Adapted from “Epicardial adipose tissue FDG uptake: Representative FDG PET/CT scan of a patient of dapagliflozin group showing SUV mean and SUV max at left circumflex artery (CX) at baseline (A) and after 4 weeks of treatment (B)” by Francesca Cinti, Lucia Leccisotti, Gian Pio Sorice, Umberto Capece, Domenico D'Amario, Margherita Lorusso, Shawn Gugliandolo, Cassandra Morciano, Andrea Guarneri, Maria Angela Guzzardi, Teresa Mezza, Amedeo Capotosti, Luca Indovina, Pietro Manuel Ferraro, Patricia Iozzo, Filippo Crea, Alessandro Giordano and Andrea Giaccari, licensed under CC BY 4.0. However, this technique is costly, has limited accessibility, and involves high radiation exposure.

Accurate detection and quantification of EAT are of considerable importance. The advantages and limitations of widely used imaging modalities are summarized in Figure 2.

EAT thickness can be evaluated via standard two-dimensional echocardiography, which is cheap, rapid, and free of radiation exposure (26). EAT is typically identified as the echo-free region located between the myocardium and pericardial visceral layer. Nevertheless, in instances of inflammation or significant accumulation of EAT, it may present as an echo-dense area (27). Although echocardiography is low-cost and readily accessible, it does not permit volumetric assessment of EAT and remains an operator-dependent technique, resulting in inter- and intra-operator variability. Cardiac multidetector CT and MRI offer precise measurements of EAT thickness and volume, and also provide additional functional information through the detection of deep regional EAT (28, 29). Compared with echocardiography, multidetector CT offers superior sensitivity and specificity for quantifying the volume and density of region-specific deep epicardial fat layers, such as peri-coronary fat, and provides detailed insights into the anatomical and pathological features of the heart (30). Fully automated deep learning algorithms using standard non-contrast CT images have recently been developed, significantly reducing the time required for EAT volume quantification compared with manual and semiautomated methods, as well as offering additional prognostic information of future adverse cardiac events (31). In addition, the average CT attenuation of EAT, particularly the perivascular fat, which is termed the perivascular fat attenuation index, has emerged as a novel imaging biomarker for coronary inflammation and a valuable tool for prognostic assessment in patients with CVDs (32, 33). Nevertheless, CT is considerably more expensive and cumbersome than echocardiographic evaluation and entails exposure to radiation. Cardiac MRI is regarded as the reference standard for evaluating total body fat distribution and visceral fat deposition (34, 35). It can provide easy assessment and volumetric quantification of EAT. Moreover, MRI avoids radiation exposure and offers high-resolution quantification. However, it remains an expensive and time-consuming modality (36).

Qualitative data on the composition of EAT can be obtained using ^18^F-fluorodeoxyglucose positron emission tomography–computed tomography (^18^F-FDG-PET-CT) (37, 38), which provides both metabolic and anatomical information. However, 18^F^-FDG-PET-CT is not considered a cost-effective approach.

## 3 Paracrine crosstalk between EAT and myocardium: a focus on EVs

EAT has been reported to contribute to the development and progression of CAD (39), increase the risk of AF (40), and predict poor prognosis in HF patients (41), particularly heart failure with preserved ejection fraction (HFpEF) (42). However, the specific molecular mechanisms connecting EAT to cardiovascular diseases remain unclear. The prevailing hypotheses involve inflammation, oxidative stress, endothelial damage, lipid accumulation, and fibrosis (4, 6).

EVs, secreted by multiple cell types, can be detected in nearly all body fluids, such as plasma, saliva, and even breast milk. They are essential for intercellular communication in multicellular organisms, facilitating the transport of molecular cargos between cells. EVs contain diverse cargos, such as lipids, proteins, and nucleic acids. They can be taken up by adjacent cells or circulated and internalized by cells to distant sites, transferring their cargos and thus participating in both physiological and pathological processes, such as cancer, diabetes, and CVDs (43).

Recently, increasing attention has been focused on the role of secretome, particularly EAT-derived EVs, in the pathogenesis of CVDs (44, 45). Given that inflammation modulation, redox balance, and fibrosis are critical processes in the development and prognosis of CVDs, characterizing the roles of EAT-derived EVs in these processes is of considerable importance and might provide valuable insights into unexplored mechanisms. Table 1 summarizes the contributions of EAT-derived EVs in the development of CVDs.

**Table 1:** EAT-derived EVs contribute to CVDs.

Effects	Diseases	Experimental model	Mediators	Ref
Inflammation modulation	CAD	EAT samples were collected from CAD patients (*n* = 24) and non-CAD patients (*n* = 15)	miR-3064-5p	(46)
	AF	EAT specimens were collected from patients with (*n* = 32) and without AF (*n* = 30)	TNF-α, IL-1α, IL-6, IL-4	(47)
Redox balance	CAD	EAT samples were collected from rat model of MI	miR-134-5p	(48)
	CAD	EAT samples were obtained from 306 patients undergoing coronary artery bypass grafting (CABG)	Adiponectin	(49)
Cardiac fibrosis	AF	EAT specimens were collected from patients with (*n* = 32) and without AF (*n* = 30)	Osteopontin, TGF-*β*	(47)
	AF	EAT specimens were collected from patients with AF (*n* = 8)	miR-1-3p, miR-133a-3p	(50)
	AF	EAT specimens were collected from patients with (*n* = 6) and without AF (*n* = 6)	hsa_cirRNA_00932	(51)
	HF	EAT samples were obtained from 39 patients undergoing CABG	Activin A, MMP8	(40)

### 3.1 Inflammation modulation

Visceral adiposity has been associated with systemic metabolic and inflammatory alterations that contribute to the development of CVDs (52). EAT functions as a bioactive organ that secretes cytokines with both anti-inflammatory and proinflammatory properties, thereby affecting adjacent tissues and contributing to chronic inflammation and myocardial damage (53, 54).

Compared with subcutaneous adipose tissue (SAT), EAT exhibits elevated expression levels of proinflammatory cytokines, such as tumor necrosis factor-α (TNF-α), interleukin-1β (IL-1β), and interleukin-6 (IL-6) (55–57). Moreover, patients with CAD display significantly higher levels of proinflammatory cytokines in EAT compared with individuals without CAD (58, 59), which are associated with the progression of CVDs and their related complications (60–62). Chang et al. demonstrated that the removal of EAT after myocardial infarction (MI) reduced the infiltration of CD45+ leukocytes and neutrophils, and decreased levels of TNF-α and IL-1β (63), indicating that EAT secretome significantly influences inflammation following MI. Huang et al. analyzed miRNA expression profiles of EAT and revealed a downregulation of miR-3624 in the EAT of CAD patients in comparison to those without CAD, which miRNA targets tumor necrosis factor receptor-associated factor 6 (TRAF6) linking with reduced inflammatory cytokine expression (64). Furthermore, EAT-derived EVs isolated from patients with CAD presented elevated expression of miR-3064-5p (46), which could induce inflammation via targeting the I*κ*B*α*/ NF-*κ*B pathway (65). Shaihov-Teper et al. isolated and purified EAT-derived EVs, and reported that EVs from patients with AF exhibited elevated levels of proinflammatory cytokines (IL-6, IL-1*α*, TNF-*α*, and IL-4) compared with those from patients without AF (47), underscoring the significant role of EAT secretome in inflammation modulation.

### 3.2 Redox balance

Myocardial redox signaling regulates several physiological processes and contributes to the pathophysiology of multiple CVDs (66). Under pathological conditions, Hao et al. demonstrated that increased levels of miR-134-5p in EAT's secretory products markedly elevated the reactive oxygen species levels, consequently promoting cardiomyocyte hypertrophy and cardiac fibroblast activation, which may lead to adverse cardiac remodeling (48). Conversely, under conditions of increased myocardial oxidative stress, antioxidant gene expression in EAT is also upregulated. Antonopoulos et al. showed that heightened myocardial oxidative stress induced increased adiponectin expression in adjacent EAT via a peroxisome proliferator-activated receptors (PPAR)-γ-dependent mechanism (49). In turn, EAT-derived adiponectin inhibited myocardial NAPDH oxidase activation, thereby protecting the heart against oxidative stress. Carena et al. reported that EAT-derived miR-92a-3p could target the Wnt5a/Rac1/NADPH oxidase axis and improve the myocardial redox state, which is negatively associated with the risk of adverse cardiovascular events (67). Together, these findings suggest that EAT can exert paracrine effects on the underlying myocardium by regulating myocardial redox homeostasis, highlighting EAT a potential therapeutic target in redox-dependent cardiovascular diseases.

### 3.3 Cardiac fibrosis

EAT-derived EVs in AF patients display a unique profibrotic profile, characterized by an enrichment of profibrotic cytokines and proteins, including osteopontin and transforming growth factor-*β* (TGF-*β*) (47). In addition, EAT-derived EVs exhibit elevated levels of miRNAs, such as miR-146b, miR-133a, and miR-29a, which are associated with collagen synthesis and atrial remodeling. Compared with EVs in SAT, altered EAT-derived EVs in patients with symptomatic, persistent, or long-standing persistent AF may impact conduction, depolarize resting potential, alter electrical cell–cell coupling, and promote arrhythmia reentry (50, 68). Zheng et al. recently identified circular RNAs from the EAT of AF patients and demonstrated that hsa_cirRNA_00932 could influence the expression of several CVD-related genes, thereby establishing a direct association between exosomal circular RNA from EAT and cardiac remodeling during the progression of AF (51). Furthermore, elevated serum levels of activin A have been reported in patients with HF (69). Venteclef et al. further demonstrated that the levels of activin A in the EAT secretome were higher in a subset of patients with left ventricular ejection fraction less than 45% (40), exerting a profibrotic effect of EAT on atrial myocardium. Other adipokines secreted by EAT, such as visfatin, TGF-*β*1, leptin, and monocyte chemoattractant protein-1 (MCP-1), have also been found to contribute to the pathogenesis of myocardial fibrosis (70).

## 4 Clinical implications

A growing body of observational evidence suggests that EAT represents a promising risk factor for CVDs. Pharmacological intervention targeting EAT could yield important cardiovascular benefits, making it a promising biomarker and therapeutic target.

### 4.1 Biomarker of CVDs

In addition to serving as an indicator of visceral adipose tissue levels, several large-scale population studies have validated the associative and predictive roles of EAT, measured by thickness or volume, in CAD development and progression (71, 72). For example, in the Heinz Nixdorf Recall cohort study, Mahabadi et al. measured EAT volume and demonstrated its association with fatal and non-fatal coronary events, independent of traditional CVD risk factors (72). Other studies have revealed a connection between increased EAT volume and the presence of high-risk coronary plaque (73, 74). Christensen et al. further showed that elevated EAT tissue levels were independently associated with an increased risk of CVDs and all-cause mortality in a prospective cohort study (75). For patients experiencing chest pain with angiographically normal coronary arteries, the measurement of EAT thickness serves as a supplementary and accessible diagnostic tool for predicting microvascular dysfunction (76). A study of patients with AF who underwent ablation therapy reported that the volume of EAT was significantly correlated with AF recurrence (77). Chu and colleagues found that greater EAT thickness was associated with increased risks of adverse cardiovascular events in AF patients, making it a useful indicator (78). In a large meta-analysis including 22 studies, increased EAT was associated with diastolic dysfunction (79). It has also been shown that EAT correlates with elevated mortality and new-onset heart failure in both HFpEF and heart failure with mildly reduced ejection fraction (80–82). ST2 is a member of the interleukin (IL)-1 receptor family with two different forms: a soluble receptor named sST2 and a transmembrane receptor named ST2L. Notably, sST2 is a potential prognostic biomarker in HF patients, independently predicting all-cause mortality and rehospitalization for HF (83, 84). Moreover, sST2 levels have been associated with EAT thickness in patients with CVDs (85, 86). Metabolic improvement and reduction in EAT are accompanied by a marked decline in sST2 concentrations, underscoring its potential as a responsive biomarker that reflects therapeutic response and adipose-driven myocardial stress (87). These findings indicate a promising translational direction where combined EAT/sST2 assessment could refine risk stratification in HF (88). However, the clinical utility of this combined approach remains investigational and requires rigorous validation in prospective interventional studies before it can be considered for routine clinical implementation.

### 4.2 Therapeutic targets in CVDs

Given its responsiveness to pharmacological agents, EAT represents a modifiable tissue whose reduction is associated with improved cardiometabolic outcomes, making it an emerging area of interest as a potential therapeutic target. Several cardiometabolic medications, including sodium-glucose cotransporter 2 (SGLT2) inhibitors, glucagon-like peptide 1 receptor (GLP1R) agonists, and statins, have demonstrated effects in reducing EAT thickness or volume. Multiple clinical studies have demonstrated significant cardiovascular benefits of these medications, although the precise underlying mechanisms have not been fully elucidated (89–94). Clinical studies evaluating the effectiveness of medications that reduce the volume or thickness of EAT are summarized in Table 2. It is critical to note that while these medications reduce EAT thickness or volume, whether this reduction is a direct mediator of their cardiovascular benefits or an effect of improved metabolic status remains to be determined.

**Table 2:** Efficacy of cardiometabolic medications in decreasing EAT thickness or volume in clinical trials.

Classification	Drug	Sample size	Population	Study design	Comparator	Imaging modality	Follow-up (weeks)	EAT change (%)	Ref.
SGLT2 inhibitors	Dapagliflozin	40	CAD	RCT	Conventional treatment	CT	24	−10	(95)
	Dapagliflozin	100	Overweight and/or obesity and T2DM	RCT	Placebo	Ultrasound	24	−20	(96)
	Canagliflozin	13	T2DM	Single-arm observational study	–	Ultrasound	24	−20	(97)
	Empagliflozin	62	HFrEF	RCT	Placebo	MRI	24	−5 mL	(98)
	Luseogliflozin	19	T2DM	Single-arm pilot study	–	MRI	12	−5	(99)
	Ipragliflozin	9	T2DM	Single-arm pilot study	–	MRI	12	−12	(100)
GLP1R agonists	Liraglutide	95	T2DM	RCT	Metformin	Ultrasound	24	−36	(101)
	Liraglutide or exenatide	25	T2DM	Single-arm observational study	–	Ultrasound	12	−13	(102)
	Liraglutide	21	T2DM	Single-arm observational	–	MRI	12	−1.6 mm	(103)
	Semaglutide	80	T2DM and obesity	Parallel group study	Metformin	Ultrasound	12	−20	(104)
	Dulaglutide	80	T2DM and obesity	Parallel group study	Metformin	Ultrasound	12	−20	(104)
Statins	Atorvastatin	420	Hyperlipidemic postmenopausal	RCT	Pravastatin	CT	48	−3.4	(105)
	Atorvastatin	145	Underwent PCI	Retrospective cohort	Simvastatin/ezetimibe	Ultrasound	24–32	−10	(106)
	Atorvastatin	79	AF	RCT	Placebo	CT	12	−6	(107)
	Pravastatin	420	Hyperlipidemic postmenopausal	RCT	Atorvastatin	CT	48	−0.8	(105)
	Simvastatin/ezetimibe	145	Underwent PCI	Retrospective cohort	Atorvastatin	Ultrasound	24–32	−3	(106)

#### 4.2.1 SGLT2 inhibitors

SGLT2, a sodium-glucose transporter that mediates renal glucose reabsorption, has been the focus of recent large-scale clinical trials. Numerous studies have revealed that SGLT2 inhibitor therapy significantly reduces major adverse cardiovascular events and cardiovascular death, demonstrating cardioprotective effects (89, 90, 108, 109). SGLT2 inhibitors, such as dapagliflozin and empagliflozin, have been shown to decrease EAT thickness or volume (95–100). Although these agents were primarily developed to lower blood glucose levels, their cardiovascular benefits may also arise from both glycosuric and non-glycemic mechanisms, including targeting EAT. For example, Díaz-Rodríguez et al. found that dapagliflozin improved EAT cell differentiation and reduced the secretion of proinflammatory chemokines in patients with CVDs (110), potentially accounting for the cardioprotective effects observed in clinical trials (109).

#### 4.2.2 GLP1R agonists

GLP1R agonists are injectable medications used for the management of type 2 diabetes and obesity, and they have demonstrated improved cardiovascular outcomes in clinical trials (92–94). In patients with type 2 diabetes and obesity, GLP1R agonists reduce EAT thickness to a greater extent than overall weight reduction (101–104). Unlike subcutaneous fat, EAT expresses GLP1R (111), enabling GLP1R agonists to exert effects on EAT. GLP1R agonists have the potential to target EAT GLP1Rs to decrease adipogenesis, enhance fat utilization, promote BAT differentiation, and modulate the renin–angiotensin–aldosterone system (112–114), thereby exhibiting cardioprotective effects.

#### 4.2.3 Statins

Statins, primarily indicated for the management of hypercholesterolemia, have been shown to reduce cardiovascular risk. Several studies have documented that stain therapy effectively reduces the quantity of EAT and decreases EAT-secreted inflammatory profiles (105–107), possibly through modulation of PPAR expression (115, 116). In patients with coronary artery stenosis, atorvastatin was associated with a notably greater reduction in EAT thickness compared with simvastatin/ezetimibe (106). Nevertheless, the effect of statins on EAT appears to be less pronounced than that of SGLT2 inhibitors and GLP1R agonists (96, 101). Notably, the combination of pioglitazone, a PPAR*γ* agonist, and simvastatin therapy has been shown to significantly reduce EAT inflammation in patients with metabolic syndrome (117).

## 5 Conclusions and perspectives

EAT is a distinct adipose depot that modulates several pathophysiological processes in the course of CVDs. It represents a quantifiable and modifiable risk factor for CVDs, providing valuable qualitative information for cardiovascular risk stratification. It is characterized as a metabolically active tissue that secretes bioactive molecules, which could be conveyed to the neighboring myocardium via paracrine mechanisms. EAT dysfunction can promote cardiomyocyte inflammation, redox imbalance, and fibrosis, subsequently affecting cardiac contractility, diastolic function, and myocardial remodeling. Characterizing the role of EAT-EVs offers insight into previously unexplored mechanisms through which EAT might promote the onset and progression of CVDs, while also identifying possible therapeutic targets. The function of EAT can be modulated and potentially restored through pharmacological interventions. Pharmacological modulation of EAT, utilizing agents such as SGLT2 inhibitors and GLP1R agonists, has been shown to decrease EAT thickness or volume and exert beneficial cardiometabolic effects.

There are several limitations to the present study. First, the causal role of EAT in CVDs remains unresolved. It is unclear whether EAT acts as a direct causal mediator, a local amplifier of systemic signals, or primarily as a marker of systemic cardiometabolic dysfunction. Mechanistic studies, particularly those elucidating the role of EAT-derived EVs, are beginning to address this question, but definitive evidence is still lacking. Second, a lack of standardization in EAT measurement across imaging modalities limits the comparability and generalizability of clinical studies. Third, while pharmacological interventions that reduce EAT are associated with improved clinical outcomes, the extent to which EAT modulation is a direct mediator of these benefits, rather than a reflection of overall metabolic improvement, has not been proven. Future research should therefore prioritize (1) establishing a consensus for EAT quantification, (2) differentiating the causal and reactive natures of EAT changes in longitudinal studies, and (3) characterizing and monitoring alterations in the EAT secretome, with particular focus on its EV cargo. Such efforts are critical to determine whether EAT represents a clinically actionable therapeutic target or a sophisticated prognostic marker. The development of novel therapies specifically designed to restore EAT's cardioprotective properties holds significant long-term promise, but remains a goal for future investigation.
